# Association Between Pro-Inflammatory Potential of Diet and Inflammatory Parameters in a Group of Patients with Inflammatory Bowel Disease

**DOI:** 10.3390/nu17172858

**Published:** 2025-09-03

**Authors:** Małgorzata Godala, Ewelina Gaszyńska, Ewa Malecka-Wojciesko

**Affiliations:** 1 Department of Nutrition and Epidemiology, Medical University of Lodz, 90-752 Lodz, Poland; ewelina.gaszynska@umed.lodz.pl; 2 Department of Digestive Tract Diseases, Medical University of Lodz, 90-419 Lodz, Poland; ewa.malecka-panas@umed.lodz.pl

**Keywords:** inflammatory bowel diseases, dietary inflammatory index, diet, inflammation, IL-6, Il-10, IL-1β

## Abstract

Background: The etiopathogenesis of IBD is not fully known; however, both genetic and environmental risk factors, including diet, are contributors to the disease. The present study aimed to determine the effect of dietary inflammatory potential, assessed using the Dietary Inflammatory Index (DII), on disease activity and inflammatory markers, such as IL-6, IL-1β, and IL-10, in patients with IBD. Methods: The study enrolled 90 patients with IBD. Dietary intake was assessed based on a 24 h questionnaire interview conducted in each subject three times. Based on these data, the DII for each subject was calculated. The serum levels of IL-6, IL-1 β, and IL-10 were determined with the quantitative sandwich enzyme-linked immunosorbent assay (ELISA). Results: The mean DII value was −0.39 ± 0.52 and did not differ significantly between the groups with CD and UC (−0.42 ± 0.47 vs. −0.37 ± 0.54, *p* = 0.6452, respectively); however, it was remarkably lower among patients in remission and with mild disease compared to those in the active phase of the disease (−0.45 ± 0.61 vs. −0.23 ± 0.65, *p* = 0.0217). Considering the DII tertiles, the subjects differed significantly in terms of age and disease activity. Logistic regression analysis of disease severity and DII in the crude model revealed that the probability of severe disease in IBD patients increased with higher DII scores. Conclusions: The results of the present study revealed a significant association between pro-inflammatory diet and IBD severity, which indicates a need to formulate an anti-inflammatory diet to reduce disease severity in patients with CD and UC.

## 1. Introduction

Inflammatory bowel disease (IBD) is an umbrella term encompassing such autoimmune disorders as ulcerative colitis (UC) and Crohn’s disease (CD). Epidemiological data indicate a rapidly increasing prevalence of these conditions worldwide. In Western Europe and North America, the incidence of UC and CD is 20 and 7 cases per 100,000 population annually, respectively. While the incidence of UC is stable, the number of CD cases has been observed to increase significantly in recent decades. In Poland, it is estimated that more than 100,000 people are diagnosed with IBD, and the condition has been more and more frequently found among very young (aged under 18) individuals [1,2,3]. The etiopathogenesis of IBD is not fully known; however, both genetic and environmental risk factors, including diet, are contributors to the disease [4,5,6,7]. As confirmed in studies, nutrients and products with pro-inflammatory properties are among the dietary factors affecting the progression and therapeutic success in IBD patients [6,7,8,9,10,11].

Although the impact of diet on health, including inflammation, is often analyzed in the literature, this issue still needs to be discussed. Attention has been paid to the role of individual nutrients in inducing and exacerbating inflammation. However, considering consumption of multiple food combinations within different eating behaviors, various authors also emphasize the importance of holistic assessment of the pro-inflammatory potential in different dietary patterns. Therefore, when determining the effect of nutrition on inflammatory processes, it is necessary to calculate the overall intake of all dietary components that may play a role in the inflammation process. In practical terms, it involves assessment of implementation of dietary standards and compliance with recommended dietary models (such as the DASH diet, Mediterranean diet, anti-inflammatory diet), or application of specially designed dietary indices developed to evaluate the pro-inflammatory potential of a diet [12,13,14,15].

One of the dietary indicators that classify different types of diet depending on their pro-inflammatory potential is the dietary inflammatory index (DII) [16,17,18,19,20,21,22]. It is based on a comprehensive review of the literature, assessed by studies on the pro-inflammatory and anti-inflammatory effects of dozens of food components, which allows for cross-national comparisons. Unlike analyses based on the assessment of specific dietary recommendations or patterns, DII is distinguished by its specific focus on the relation between dietary intake and the onset and exacerbation of inflammation. Hence, it is reasonable to evaluate this relation in IBD patients.

The association between DII and inflammatory markers has been demonstrated both in studies involving groups of patients treated for various conditions and the general population. In these studies, the DII has been found to be useful in predicting inflammatory parameters such as levels of C-reactive protein (CRP), interleukin-6 (IL-6) or adiponectin [15,23,24]. However, there are few studies on the pro-inflammatory potential of diet assessed based on dietary indicators and their impact on IBD activity, and the results are inconclusive. Some authors have shown an association between a diet rich in inflammatory factors and increased IBD risk and disease activity [13,16,18,25,26,27,28,29]. Whereas other researchers have not confirmed such a correlation, indicating that the dietary pro-inflammatory potential has no effect on the course of IBD [30,31].

The present study aimed to determine the effect of dietary inflammatory potential, assessed using DII, on disease activity and inflammatory markers, such as IL-6, IL-1β and IL-10, in patients with IBD.

## 2. Materials and Methods

### 2.1. Study Participants

The study enrolled 90 patients with IBD, including 46 individuals with CD and 44 with UC, treated at the Department of Digestive Tract Diseases, Medical University of Lodz. The exclusion criteria for the study were presence of cancer, metabolic and cardiovascular diseases. Disease activity was assessed using the Crohn’s Disease Activity Index (CDAI) for CD patients and the Partial Mayo Score for UC patients [32,33]. According to the guidelines of the European Crohn’s and Colitis Organization (ECCO), CDAI < 150 was defined as disease remission, CDAI 150–220 as a mild exacerbation of symptoms and CDAI 220–450 as a moderate exacerbation of symptoms, while CDAI > 450 was considered a severe exacerbation of disease symptoms. A Partial Mayo Score of 0 corresponded to clinical remission, while mild activity of the disease was diagnosed for a Partial Mayo Score of 1, moderate for a Partial Mayo Score of 2 and severe for a Partial Mayo Score of 3 [32,33].

All the subjects were measured for height and weight to calculate BMI (Body Mass Index). Malnutrition was diagnosed for BMI < 18.5 kg/m^2^, normal nutritional status for BMI from 18.5 to 24.9 kg/m^2^, overweight for BMI from 25.0 to 29.9 kg/m^2^, and obesity for BMI from 30.0 kg/m^2^ [34]. Also, body composition was estimated in the study participants with the Bioelectrical Impedance Analysis (BIA) method using an InBody 270 apparatus (Seoul, Republic of Korea). Body fat (Fatt Mass; FM) and fat-free mass (FFM) were measured.

### 2.2. Blood Analysis

Blood samples for laboratory tests were collected fasting from the ulnar vein first day of hospital admission and then centrifuged (2000× *g* for 20 min). The isolated serum was frozen at −80 °C. The obtained samples were used to determine inflammatory markers.

The serum levels of IL-6, IL-1 β, IL-10 were determined with the quantitative sandwich enzyme-linked immunosorbent assay (ELISA), using kits from Biorbyt LLC, Durham, NC, USA. All tests were performed according to manufacturer’s instructions.

### 2.3. Dietary Assessment

Dietary intake was assessed based on a 24 h questionnaire interview conducted in each subject three times (two days of work activity and one holiday), visual aids were used (food photography) to estimate portion size. The mean intake of energy and nutrients was calculated using the computer program Diet 6.0 (license No. 52/PD/2022). Additionally, based on the Food Frequency Questionnaire (FFQ), the frequency of consumption of selected food groups was evaluated qualitatively and quantitatively in the last month prior to the study. It assessed intake of fruits, vegetables, dry seeds of legumes, whole grain products, refined flour products, red meat, poultry, fish, dairy products, butter, vegetable oils, fast-food products, sweetened beverages, sweets, coffee and tea [35].

Based on these data and using the method proposed by Shivappa et al., DII for each subject was calculated. The daily intake of 26 nutritional parameters was assessed, i.e., energy, protein, carbohydrates, total fat, monounsaturated fatty acids, polyunsaturated fatty acids, cholesterol, fiber, micronutrients (zinc, iron, magnesium, selenium, vitamins A, B1, B12, B3, B6, C, D, E, and folic acid), and selected flavonoids (anthocyanins, flavones, flavonols, flavanols, flavonones) [36]. The z-score was calculated and converted into a centered percentile score. The resulting value was multiplied by the inflammatory score of each dietary component, and then the individual global inflammatory index was calculated by adding up all DII scores specific to the assessed food parameters. Negative values indicated an anti-inflammatory diet, while positive values indicated a pro-inflammatory dietary pattern.

### 2.4. Statistical Analysis

All data were analyzed using Statistica™ 14 (TIBCO Software Inc., Palo Alto, CA, USA). The obtained results were presented as the mean and standard deviation for quantitative variables, and as numerical values and percentages for qualitative variables. The normality of the distribution was assessed using the Shapiro–Wilk test. In the case of univariate analysis, the Mann–Whitney U test was used when the grouping variable was dichotomous, and the Kruskal–Wallis H test when the grouping variable had more than two categories.

The obtained DII score was divided into tertiles. ANOVA was used to measure differences in numerical data within the tertiles. The relationship between disease severity and DII score tertiles was analyzed using logistic regression. A value of *p* < 0.05 was considered statistically significant.

The study was conducted in accordance with the Declaration of Helsinki and approved by the Bioethics Committee of the Medical University of Lodz (No. RNN/70/22/KE, 14 June 2022). All the subjects gave written consent to participate in the study.

## 3. Results

The study enrolled 90 patients with IBD, including 52 women (57.8%) and 38 men (42.2%). The mean disease duration was 8.2 ± 5.6 years. Most of the study participants received biological treatment (82.2%). Among the IBD group, 32 individuals (35.6%) were in clinical and endoscopic remission, 20 (22.2%) had mild disease, 28 (31.1%) moderate disease, and 10 (11.1%) severe disease. Nearly one in four subjects smoked cigarettes (23.3%) (Table 1).

The mean DII value was −0.39 ± 0.52 and did not differ significantly between the groups with CD and UC (−0.42 ± 0.47 vs. −0.37 ± 0.54, *p* = 0.6452, respectively); however, it was remarkably lower among patients in remission and with mild disease compared to those in the active phase of the disease (−0.45 ± 0.61 vs. −0.23 ± 0.65, *p* = 0.0217). The mean consumption of the analyzed food products according to the DII value is presented in Table 2.

Considering the DII tertiles, the subjects differed significantly in terms of age and disease activity. No significant differences were found in terms of gender, disease duration, BMI, smoking, or type of treatment received. Moreover, no significant differences were observed in IL-6 and IL-1β levels in relation to DII scores. As for IL-10 levels depending on the DII tertiles, there were no significant differences found either (Table 3).

Logistic regression analysis of disease severity and DII in the crude model revealed that the probability of severe disease in IBD patients increased with higher DII scores. In both adjusted models, we demonstrated a significant association between disease severity and DII score tertiles. The final model showed that the probability of severe disease course was three times higher in patients on a pro-inflammatory diet compared to those on an anti-inflammatory diet [OR: 3.13 (95% CI: 1.11; 9.51, *p* = 0.0017] (Table 4).

## 4. Discussion

Inflammation, which exacerbates the symptoms of IBD, can be modulated by nutritional parameters [37,38,39]. Therefore, diet can impact the progression and severity of IBD by affecting inflammation [38]. The present study assessed the association between a pro-inflammatory diet and IBD activity using the DII score. The DII, based mainly on dietary components, was developed using the data documented in studies on the association between food products and nutrients and inflammatory markers [36]. The results of our study showed that the mean DII score was significantly higher in patients with active disease compared to those in remission or with mild IBD. This was also reflected in the results of regression analysis. After taking into account factors such as BMI, gender, disease duration, biological treatment, and smoking, the analysis proved that the probability of disease activity was significantly higher in patients on a pro-inflammatory diet than among those on an anti-inflammatory diet.

Similar data were obtained in other research. A study by Keshteli et al. revealed that increased consumption of anti-inflammatory products and lower consumption of pro-inflammatory food effectively alleviated symptoms in patients with IBD [28]. Also, a study by Rocha et al. demonstrated a relationship between a DII score and disease activity in individuals with IBD [29]. An analysis of three prospective cohort studies (Nurses’ Health Study, Nurses’ Health Study II, Health Professionals Follow-up Study) showed that dietary patterns with high inflammatory potential were associated with an increased risk of CD and UC [40].

However, contrasting data were obtained in a study by Mirmiran et al., in which the authors did not confirm any relationship between the pro-inflammatory potential of diet and disease activity [31]. When assessing the impact of pro-inflammatory potential of diet in a group of UC patients, Lamers et al. also showed that the pro-inflammatory value of diet, assessed using DII, was not associated with disease activity [30]. The conflicting findings obtained in various studies may result from different methods of assessing disease activity and pro-inflammatory potential of diet, such as the number and types of food products analyzed to calculate the DII score. The discrepancies may also be due to a small sample size or the fact that most subjects followed an anti-inflammatory diet, with a low proportion of patients eating a pro-inflammatory diet. Additionally, in the cited studies, patients were supplemented with n-3 fatty acids. Clinical studies suggest that n-3 fatty acid supplementation in patients with IBD reduces inflammation [41].

A meta-analysis conducted in 2025 reveals that the inflammatory potential of diet, assessed using the DII, is associated with IBD activity. The assessment of the risk of IBD development showed contradictory results, which may be associated with differences in methodology. This suggests that evaluating the inflammatory potential of diet may serve as a tool in the treatment of IBD; however, its usefulness in preventing the disease remains unclear [42].

Studies have shown that diet is an important moderator of inflammation, and specific foods, nutritional and bioactive components of diet may produce immunomodulatory effects [16,18,22]. Studies indicate that consumption of fruits and vegetables, herbs and spices, fiber, magnesium, n-3 PUFA, MUFA, flavonoids and carotenoids from food products are associated with reduced serum levels of inflammatory markers. On the other hand, saturated fatty acids (SFA), high-glycemic (GI) carbohydrates and high n-6 to n-3 ratio in the diet are associated with increased levels of inflammation [18,22,28]. In the present study, we did not confirm the association between the pro-inflammatory potential of diet, assessed based on DII, and increased levels of inflammatory cytokines, such as IL-6 and IL-1β, in IBD patients. The literature includes a large number of studies confirming the relationship between the pro-inflammatory potential of diet and levels of inflammatory markers in various groups of patients and general populations [20,43,44]. However, there are no studies available that evaluate the association of DII with levels of inflammatory markers in IBD patients.

The lack of correlation between DII scores and levels of inflammatory cytokines in the present study can be explained by several factors. Patients with IBD tend to change their diet, consciously abstaining from certain foods for fear of aggravation of symptoms [45]. Despite this belief, there is limited evidence that proves the relationship between dietary components and disease activity in these patients [46,47,48]. In our study, blood samples for laboratory tests were collected from IBD patients only once, therefore it was not possible to assess long-term diet-dependent changes. This limited the ability to reliably evaluate the relationship between inflammatory potential of diet and inflammatory markers. Additionally, the DII was developed based on the effect of dietary factors on six inflammatory markers [36]. In the present study, we verified only three inflammatory cytokines, which may explain the lack of correlation between DII and levels of the interleukins studied.

This study has some limitations. Due to its cross-sectional nature, causal inference is restricted. Moreover, it is a single-center study conducted in a small group of patients, which makes it difficult to apply the results to the whole population of IBD patients. To assess the intake of energy and nutrients as well as food groups, a 24 h interview and FFQ were used, therefore certain limitations cannot be excluded (e.g., inaccurate assessment of the amount of consumed food and memory gaps). Another important limitation of our study is that the DII has not been validated using inflammatory markers in the Polish population. Moreover, the study included individuals who were aware of their disease and probably modified their diet after diagnosis, which may lead to measurement error. Finally, although the association of DII and IBD severity was adjusted for various confounding variables, some unidentified and residual co-occurring variables could not be controlled.

## 5. Conclusions

The results of the present study revealed a significant association between pro-inflammatory diet and IBD severity, which indicates a need to formulate an anti-inflammatory diet to reduce disease severity in patients with CD and UC. Considering the potential limitations of this study, further research is required to confirm the hypothesis that diet-induced inflammation may affect disease activity in patients with IBD.

## Figures and Tables

**Table 1:** General characteristics of study participants.

	IBD *n*(%), Mean ± SD	CD *n*(%), Mean ± SD	UC *n*(%), Mean ± SD	*p*
Patients	90 (100)	46 (51.1)	44 (48.9)	0.1278
Age [years]	36.9 ± 9.7	33.8 ± 9.4	42.6 ± 10.7	0.0010
Female	52 (57.8)	28 (60.9)	24 (54.5)	0.1057
Duration of the disease [years]	8.2 ± 5.6	8.1 ± 5.4	8.2 ± 4.6	0.7234
CDAI (0/1/2/3)		15 (32.6)/10 (21.7)/17 (37.0)/4 (8.7)		
Partial Mayo Score (0/1/2/3)			17 (38.6)/10 (22.7)/11 (25)/6 (13.6)	
Biological therapy	74 (82.2)	41 (89.1)	33 (75)	0.3125
Smokers	21 (23.3)	10 (21.7)	11 (25)	0.6784
BMI [kg/m^2^]	24.2 ± 4.6	23.7 ± 4.1	24.5 ± 4.3	0.4705
≤18.5	20 (22.2)	10 (21.7)	10 (22.7)	0.6545
18.5–24.9	36 (40)	20 (43.5)	16 (36.4)	
≥25.0	34 (37.8)	16 (34.8)	18 (40.9)	
IL-6 [pg/mL]	5.3 ± 1.8	5.1 ± 1.7	6.1 ± 1.5	0.2178
IL-1β [pg/mL]	7.2 ± 1.8	7.4 ± 1.9	6.9 ± 2.2	0.2566
IL-10 [pg/mL]	8.9 ± 1.8	7.9 ± 1.9	9.3 ± 2.1	0.5122
Dietary assessment				
DII	−0.39 ± 0.52	−0.42 ± 0.47	−0.37 ± 0.54	0.6452
Energy intake [kcal]	1565 ± 290	1534 ± 287	1586 ± 321	0.7658
Calories from carbohydrates [% E]	52.1 ± 6.3	53.1 ± 6.1	52.8 ± 6.6	
Calories from fats [% E]	26.9 ± 6.2	26.6 ± 7.1	27.7 ± 5.4	0.3725
Calories from proteins [% E]	18.1 ± 3.7	18.1 ± 4.2	18.1 ± 3.1	0.6034
Fat [g]	41.7 ± 8.9	40.5 ± 9.1	43.8 ± 8.7	0.4335
SFA [g]	17.8 ± 1.6	17.2 ± 1.7	18.1 ± 1.7	0.6785
MUFA [g]	14.1 ± 2.7	13.7 ± 3.3	14.7 ± 2.1	0.4781
PUFA [g]	7.1 ± 2.1	7.2 ± 2.2	7.1 ± 1.8	0.4776
Cholesterol [mg]	211 ± 15.7	212.6 ± 15.9	210.7 ± 15.6	0.3132
Protein [g]	67.8 ± 9.1	65.4 ± 8.9	71.7 ± 9.2 *	0.0231
Fiber [g]	14.2 ± 3.5	9.7 ± 4.5	16.9 ± 3.7 *	0.0227
Carbohydrates [g]	237.7 ±	235.5 ± 80.4	242.8 ± 72.3	0.3234
Simple sugars [g]	10.7 ± 3.8	11.5 ± 4.1	9.8 ± 3.3	0.6543
Dietary folate equivalents [μg]	211.5 ± 40.9	212.1 ± 39.1	210.5 ± 42.4	0.4137
Vitamin A [μg retinol equivalent]	1278.7 ± 280.9	1255.4 ± 310.1	1307.3 ± 270.5	0.7651
Vitamin E [mg α-tokoferol equivalent]	5.3 ± 2.0	5.4 ± 2.1	5.3 ± 1.8	0.6743
Vitamin C [mg]	74.1 ± 35.1	72.7 ± 34.1	76.1 ± 36.2	0.4556
Vitamin B-12 [μg]	2.1 ± 0.6	2.1 ± 0.5	2.2 ± 0.8	0.3045
Vitamin D [μg]	2.4 ± 1.3	2.3 ± 1.2	2.5 ± 1.6	0.5484
Calcium [mg]	528.4 ± 100.4	522.1 ± 90.2	531.4 ± 110.6	0.5905
Magnesium [mg]	305.6 ± 89.7	299.3 ± 88.7	310.3 ± 90.3	0.4825
Iron [mg]	8.6 ± 2.9	8.7 ± 3.1	8.3 ± 2.9	0.2384
Zinc [mg]	7.9 ± 2.5	7.7 ± 2.1	8.1 ± 3.1	0.3705
Sodium [μg]	2875.8 ± 260.8	3074.5 ± 210.1	2779.8 ± 310.2	0.4225
Potassium [mg]	2501.5 ± 178.6	2553.1 ± 216.5	2468.5 ± 122.7	0.5672

DII: dietary inflammatory index; SFA: saturated fatty acid; MUFA: monounsaturated fatty acid; PUFA: polyunsaturated fatty acid; * *p*-value of one-way ANOVA.

**Table 2:** Dietary intake of patients with IBD according to DII tertiles.

Variables	DII			*p*-Value
	T1 (−2.34; −0.61)	T2 (−0.61; 0.27)	T3 (0.27; 1.74)	
Energy intake [kcal]	1698.1 ± 371.6	1485.2 ± 333.8	1429.2 ± 339.1	0.163
Protein [g]	70.3 ± 13.1	67.8 ± 8.5	65.2 ± 11.5	0.042
Carbohydrate [g]	251.4 ± 70.9	237.3 ± 66.2	228.4 ± 74.8	0.451
Fat [g]	52.9 ± 12.7	47.1 ± 9.9	40.9 ± 13.1	0.011
Cholesterol [mg]	230.8 ± 50.8	220.2 ± 75.8	210.9 ± 59.98	0.311
SFA [g]	17.9 ± 4.9	17.2 ± 3.5	17.4 ± 5.6	0.872
MUFA [g]	18.5 ± 5.5	16.6 ± 6.4	15.5 ± 7.5	0.142
PUFA [g]	8.94 ± 2.5	5.9 ± 3.4	4.37 ± 2.8	0.011
Fiber [g]	19.5 ± 3.6	16.5 ± 2.8	14.1 ± 2.7	0.012
Beta Carotene [µg]	720.9 ± 313.1	439.5 ± 322.5	282.8 ± 271.9	0.011
Vitamin A [μg retinol equivalent]	1572.8 ± 560.3	909.4 ± 408.4	780.6 ± 343.3	0.014
Vitamin B1 [mg]	2.0 ± 0.4	2.0 ± 0.3	1.99 ± 0.5	0.331
Vitamin B2 [mg]	1.5 ± 0.3	1.3 ± 0.3	1.2 ± 0.3	0.005
Vitamin B3 [mg]	20.9 ± 4.4	20.6 ± 4.2	19.5 ± 5.5	0.681
Vitamin B6 [mg]	1.9 ± 0.8	1.7 ± 2.3	0.9 ± 0.2	0.001
Dietary folate equivalents [μg]	0.8 ± 0.3	0.9 ± 0.2	0.8 ± 0.3	0.081
Vitamin B12 [µg]	3.6 ± 1.5	2.8 ± 1.3	2.3 ± 1.3	0.001
Vitamin C [mg]	92.4 ± 33.3	63.5 ± 19.4	47.9 ± 17.3	0.001
Vitamin D [µg]	2.3 ± 1.2	2.1 ± 0.9	2.3 ± 1.2	0.123
Vitamin E [mg α-tokoferol equivalent]	5.3 ± 3.88	4.0 ± 1.5	3.2 ± 1.5	0.002
Caffeine [g]	190.9 ± 93.9	185.1 ± 93.1	252.3 ± 120.3	0.011
Zinc [mg]	7.3 ± 1.3	6.4 ± 1.4	5.4 ± 1.4	0.001
Selenium [µg]	120.0 ± 3.1	100.3 ± 3.4	80.8 ± 2.9	0.001
Magnesium [mg]	239.3 ± 52.1	199.2 ± 36.7	181.5 ± 52.8	0.001
Iron [mg]	16.1 ± 3.3	14.6 ± 2.7	13.3 ± 3.4	0.005
Onion [g]	9.3 ± 18.2	8.9 ± 12.4	9.2 ± 14.5	0.991
Garlic [g]	0.8 ± 0.6	0.3 ± 0.4	0.5 ± 0.9	0.259
Green/black tea [g]	584.9 ± 510.4	711.9 ± 471.3	1043.2 ± 434.2	0.012

DII: dietary inflammatory index; SFA: saturated fatty acid; MUFA: monounsaturated fatty acid; PUFA: polyunsaturated fatty acid.

**Table 3:** Characteristics of study participants according to DII tertiles.

	DII Tertiles			*p*-Value
	T1 (−2.34; −0.61)	T2 (−0.61; 0.27)	T3 (0.27; 1.74)	
Age [years]	30.1 ± 8.7	35.5 ± 9.9	40.1 ± 9.8	0.0213
Sex				0.6574
Female	16 (57.1)	18 (56.3)	17 (56.7)	
Male	12 (42.9)	14 (43.7)	13 (43.3)	
Disease duration [years]	8.1 ± 5.5	7.9 ± 5.3	8.7 ± 5.7	0.3487
Disease activity				0.0382
Remission and mild stage	23 (82.1)	19 (59.4)	10 (33.3)	
Moderate and severe stage	5 (17.9)	13 (40.6)	20 (66.7)	
Smokers	6 (21.4)	8 (25)	7 (23.3)	0.2381
Biological treatment	23 (82.1)	26 (81.3)	25 (83.3)	0.6473
BMI [kg/m^2^]	23.7 ± 5.6	24.3 ± 4.7	25.3 ± 4.1	0.2361
IL-6 [pg/mL]	5.3 ± 1.9	5.2 ± 1.5	6.1 ± 1.9	0.1218
IL-1β [pg/mL]	4.9 ± 1.3	7.2 ± 1.8	6.9 ± 1.9	0.2116
IL-10 [pg/mL]	8.9 ± 1.5	7.7 ± 1.8	10.3 ± 2.2	0.5632

BMI: body mass index; T: tertile; DII: Dietary inflammatory index.

**Table 4:** Logistic regression analysis for IBD severity according to tertiles of DII score.

	DII Categories			DII (Continuous)	
	T1 (*n* = 28)	T2 (*n* = 32)	T3 (*n* = 30)		
		OR (95% CI)	OR (95% CI)	OR (95% CI)	*p*-Value ***
Crude model	1 (Ref)	2.76 (1.15; 6.85)	2.41 (0.98; 5.58)	1.73 (1.21; 2.74)	0.031
Model 1 *	1 (Ref)	3.14 (1.16; 8.56)	2.75 (1.15; 6.80)	1.84 (1.20; 3.21)	0.022
Model 2 **	1 (Ref)	3.49 (1.19; 8.04)	3.13 (1.11; 9.51)	2.25 (1.23; 4.26)	0.017

DII: dietary inflammatory index; OR: odds ratio; CI: confidence interval; T: tertile. Dependent factor: disease severity; independent variable: tertiles of DII. Disease severity was categorized into remission and mild active (Partial Mayo Score and CDAI 0-1), and moderately and severe active disease (Partial Mayo Score and CDAI 2-3); * Adjusted for body mass index and biological treatment. ** Adjusted for model 1 plus age, sex, smoking, and disease duration. *** *p*-value of trend.

## Data Availability

The original contributions presented in this study are included in the article. Further inquiries can be directed to the corresponding author(s).
